# Paracetamol: A History of Assumptions, Errors, and Evidence Reversals in Pain Management

**DOI:** 10.1007/s11916-026-01553-w

**Published:** 2026-09-12

**Authors:** Jessica Nicolas, Matteo Luigi Giuseppe Leoni, Y Van Tran, Pham Van Phong, Giacomo Farì, Piercarlo Sarzi Puttini, Alan D. Kaye, Giustino Varrassi

**Affiliations:** 1 Chronic Pain Management Center, Paris Saint-Joseph Hospital, Paris, France; 2 Visionary International Board for Research and Analgesia (VIBRA), Fondazione Paolo Procacci, Rome, 00193 Italy; 3 Rheumatology Unit, IRCCS Ospedale Galeazzi Sant’Ambrogio, Milan, Italy; 4 Department of Biomedical and Clinical Sciences, University of Milan, Milan, Italy; 5 Department of Medical and Surgical Sciences and Translational Medicine, Sapienza University of Rome, Rome, Italy; 6 Department of Anesthesiology, Tam Anh General hospital, Ho Chi Minh City, 700000 Vietnam; 7 Department of Biomedical Engineering, International University, Ho Chi Minh City, 70000 Vietnam; 8 Department of Experimental Medicine (Di.Me.S.), University of Salento, Lecce, Italy; 9 Department of Anesthesiology, Louisiana State University Health Sciences Center, Shreveport,, Louisiana 71103 USA; 10 College of Medicine, University of Baghdad, Baghdad, Iraq

**Keywords:** Acetaminophen, Paracetamol, Analgesics, Evidence reversal, Hepatotoxicity, Pain management, History of medicine, Therapeutic inertia

## Abstract

**Purpose of Review:**

Paracetamol (acetaminophen) is among the most widely used medications in the history of humanity for treatment of pain conditions. For more than half a century, it has occupied a unique position as the archetypal first-line analgesic and antipyretic, recommended across age groups, clinical settings, and healthcare systems. Its remarkable success has been based on three fundamental assumptions: it is effective for common painful conditions, substantially safer than alternative analgesics, and suitable for broad population use with minimal restrictions. Over the last two decades, however, these assumptions have been increasingly challenged by accumulating evidence from randomized trials, systematic reviews, pharmaco-epidemiological studies, and regulatory analyses.

**Recent Findings:**

The history of paracetamol represents an instructive example of evidence reversal in modern medicine and pain management. Originally introduced as a safer successor to phenacetin, paracetamol benefited from a favorable safety reputation that often exceeded the available scientific evidence. Subsequent investigations have revealed a more complex reality. Hepatotoxicity has emerged as a leading cause of acute liver failure worldwide, efficacy in chronic musculoskeletal disorders has proved substantially lower than previously assumed, and concerns regarding long-term safety and use during pregnancy have generated ongoing scientific debate. Simultaneously, clinical practice and prescribing habits remained largely unchanged despite evolving evidence and repeated modifications of international guidelines.

**Summary:**

By examining major assumptions, scientific misconceptions, regulatory decisions, and evidence reversals, this review explores how one of medicine’s most familiar drugs in pain medicine became a paradigm of the challenges inherent in translating evolving scientific evidence into clinical practice. Beyond paracetamol itself, this story provides broader lessons regarding therapeutic inertia, medical dogma, risk perception, and the dynamic nature of evidence-based medicine.

**Supplementary Information:**

The online version contains supplementary material available at https://doi.org/10.1007/s11916-026-01553-w.

## Introduction

Few drugs have achieved the cultural, clinical, and commercial prominence of paracetamol in pain medicine. Known as acetaminophen in North America and paracetamol throughout most of the world, the compound has become synonymous with safe pain relief [1]. It is recommended for fever, acute pain, chronic pain, postoperative recovery, musculoskeletal disorders, pregnancy-associated discomfort, pediatric illnesses, and palliative care [2]. Global consumption is measured in tens of billions of doses annually, making it one of the most frequently used medications worldwide [3].

Yet the history of paracetamol is neither straightforward nor uniformly successful. Beneath its reputation as a benign and universally useful analgesic lies a more complex narrative marked by scientific uncertainty, evolving mechanisms of action, changing safety perceptions, and repeated reassessments of clinical value [4]. The journey of paracetamol illustrates how therapeutic beliefs can become established long before robust evidence becomes available and how such beliefs may persist even after the emergence of contradictory data.

The history of paracetamol began largely by accident. Synthesized during the nineteenth century, the compound remained neglected for decades while phenacetin and acetanilide dominated the analgesic market to treat pain conditions. Only after concerns regarding the toxicity of these agents emerged did paracetamol gain prominence as their apparently safer successor [5, 6]. During the latter half of the twentieth century, the drug became embedded in clinical practice as the preferred first-line analgesic, particularly because it appeared to avoid the gastrointestinal toxicity associated with nonsteroidal anti-inflammatory drugs (NSAIDs) and the dependence potential associated with opioids [7]. For decades, this favorable perception remained largely unquestioned. Paracetamol was considered effective, safe, inexpensive, and universally applicable.

However, the scientific foundations supporting these assumptions were often less robust than commonly appreciated. Many therapeutic recommendations were based on extrapolation, tradition, expert opinion, or relatively limited clinical evidence [5, 8]. As methodologies in clinical research improved and large randomized controlled trials became more common, long-standing assumptions were increasingly subjected to rigorous scrutiny [9].

The last two decades have witnessed what may be described as an evidence-based reappraisal of paracetamol. Randomized trials and meta-analyses have demonstrated limited efficacy in several chronic pain conditions for which the drug had been routinely recommended [8–10]. These findings prompted substantial reconsideration of long-standing therapeutic recommendations and contributed to revisions of multiple international clinical practice guidelines [10, 11].

At the same time, safety concerns evolved considerably. Although still safer than many alternatives in numerous clinical contexts, paracetamol proved far from harmless. Intentional and unintentional overdose emerged as a leading cause of acute liver failure in many countries and remains one of the most common causes of drug-induced liver injury worldwide (12–14]. Questions were subsequently raised regarding cardiovascular, renal, and developmental outcomes associated with prolonged exposure, particularly in vulnerable populations and with chronic use [15, 16]. Regulatory agencies responded by introducing restrictions on prescription combination products, limiting paracetamol content per dosage unit, and strengthening warnings regarding dosage thresholds and hepatotoxicity [13, 17].

Interestingly, these developments occurred without a proportional reduction in the drug’s use. Paracetamol continues to occupy a privileged position within healthcare systems despite growing uncertainty regarding its efficacy in many chronic pain conditions [2, 9, 18]. Real-world studies have shown that paracetamol remains one of the most prescribed first-line analgesics for low back pain and osteoarthritis, even after major randomized trials and meta-analyses questioned its clinical benefit [9, 18].

The purpose of this narrative review, therefore, is not to condemn paracetamol nor to advocate for its abandonment in pain management. Rather, it is to critically examine its historical trajectory through the lens of evidence evolution. By revisiting the origins of the drug, the assumptions that supported its widespread adoption, the errors and misconceptions that shaped its reputation, and the evidence reversals that transformed contemporary understanding, this review seeks to provide a balanced perspective on one of the most influential medications in modern medicine. Specifically, although the broad outline of paracetamol’s troubled history has been described previously [5], the present review extends earlier accounts by integrating the most recent efficacy trials and dose-response safety data into a single interpretative framework and by using this trajectory to derive transferable lessons for the evaluation of other long-established therapies.

## Methods

### Study Design

The present investigation was conceived as a structured historical narrative review aimed at critically examining the evolution of scientific knowledge surrounding paracetamol, with particular emphasis on the assumptions, misconceptions, evidence reversals, and regulatory decisions that have shaped its clinical use over more than a century. The review was conducted and reported in accordance with the principles of the Scale for the Assessment of Narrative Review Articles (SANRA), which provides a validated framework for ensuring methodological transparency, scientific rigor, and balanced interpretation in narrative reviews [19].

Unlike systematic reviews, which are designed to answer narrowly defined clinical questions through exhaustive evidence synthesis, the present review adopted a broader historical and interpretative perspective. The objective was not merely to summarize the available literature but to reconstruct the scientific trajectory of paracetamol, tracing the interplay between pharmacological discovery, clinical adoption, safety perceptions, evolving evidence, and healthcare policy. Particular attention was devoted to identifying pivotal events and publications that contributed to major shifts in scientific understanding and clinical practice.

## Literature Search Strategy

A comprehensive literature search was performed using PubMed/MEDLINE, Scopus, Web of Science, and Embase. Searches were conducted from database inception through June 1, 2026. Additional sources were identified through manual examination of reference lists from relevant reviews, historical publications, regulatory documents, clinical practice guidelines, and seminal articles frequently cited within the paracetamol literature.

The search strategy was developed to capture the multifaceted evolution of paracetamol from its historical origins to its contemporary clinical and regulatory reassessment. Accordingly, combinations of controlled vocabulary terms and free-text keywords were used to identify literature addressing the discovery and development of paracetamol and related compounds, including phenacetin and acetanilide, as well as its pharmacological properties, clinical applications, efficacy, safety profile, and regulatory history. Particular attention was devoted to publications exploring major themes that have shaped the scientific narrative of paracetamol, including analgesic efficacy, pain management, hepatotoxicity, acute liver failure, overdose, drug regulation, clinical practice recommendations, guideline development, evidence reversal, and emerging concerns related to pregnancy and neurodevelopmental outcomes. The search strategy was iteratively refined throughout the review process to ensure comprehensive coverage of both historical milestones and contemporary evidence.

The search strategy was intentionally designed to capture not only clinical efficacy and safety studies but also historical analyses, pharmacological investigations, regulatory communications, policy documents, and publications addressing broader conceptual issues related to evidence-based medicine and therapeutic decision-making.

## Selection of Sources

Given the historical and interpretative nature of this review, the selection of sources was guided by relevance and scholarly significance rather than by the exhaustive retrieval principles typically applied to systematic reviews. Priority was assigned to publications that had substantially influenced the development of paracetamol as a therapeutic agent, advanced understanding of its pharmacological mechanisms, shaped clinical practice through landmark trials or meta-analyses, informed regulatory policies, or contributed to major shifts in scientific consensus. Contemporary reviews and guideline documents were also examined to contextualize current perspectives on efficacy, safety, and clinical use. To preserve historical continuity and accurately reconstruct the evolution of scientific knowledge, both classical and recent references were included. Older publications were retained when they represented seminal milestones in the history of paracetamol or provided unique insights that remain relevant to contemporary interpretation. To enhance methodological strength and minimize the risk of selection bias, two reviewers independently conducted the screening and eligibility assessment. Any disagreements on study inclusion were addressed through discussion until consensus was reached. If consensus could not be obtained, a third senior reviewer independently assessed the study and made the final decision.

## Data Extraction and Thematic Synthesis

Relevant publications were reviewed in full text and analyzed qualitatively. Information was extracted regarding historical developments, pharmacological discoveries, clinical assumptions, efficacy findings, safety signals, regulatory interventions, guideline modifications, and changes in therapeutic positioning over time.

The synthesis was organized according to a chronological-conceptual framework that reflected major phases in the scientific history of paracetamol. These phases included:

1. Discovery and early pharmacological development.
2. Emergence as a successor to acetanilide and phenacetin.
3. Establishment of the “safe analgesic” paradigm.
4. Evolution of mechanistic understanding.
5. Reassessment of efficacy in chronic pain conditions.
6. Emergence of safety concerns beyond hepatotoxicity.
7. Regulatory responses and policy changes.
8. Persistence of clinical use despite evolving evidence.

Rather than focusing exclusively on individual study outcomes, the review emphasized patterns of evidence accumulation, areas of consensus and controversy, and the mechanisms through which scientific assumptions became established, challenged, or revised over time.

## Critical Appraisal and Interpretative Framework

Formal risk-of-bias assessment was not performed because the objective of the review was not quantitative evidence synthesis. Instead, a critical interpretative approach was adopted. Greater weight was assigned to landmark randomized controlled trials, systematic reviews, meta-analyses, major regulatory reports, and authoritative clinical guidelines. Historical publications were evaluated according to their influence on subsequent scientific developments and their role in shaping clinical perceptions.

The interpretative framework was informed by concepts derived from the history and philosophy of medicine, particularly the notions of evidence evolution, therapeutic inertia, cognitive bias in clinical decision-making, and evidence reversal. This approach allowed the review to move beyond a descriptive chronology and to examine how scientific knowledge surrounding paracetamol evolved in response to changing methodological standards and emerging evidence.

## Results

The historical trajectory of paracetamol has been largely shaped by three interrelated assumptions that supported its widespread acceptance in clinical practice: that it is an effective analgesic for common painful conditions, that it is safer than alternative analgesics, and that it can be used broadly across diverse patient populations with minimal restrictions. These assumptions were established over decades through a combination of early clinical experience, extrapolation from acute pain models, favorable comparisons with NSAIDs and opioids, and endorsement by clinical guidelines. However, the evidence accumulated during the last two decades has prompted a substantial reassessment of each of these paradigms.

Systematic reviews, large observational studies, pharmacovigilance analyses, and regulatory evaluations have progressively challenged the magnitude of paracetamol’s efficacy in several chronic pain conditions, highlighted safety concerns extending beyond hepatotoxicity, and questioned the appropriateness of its indiscriminate use across all patient groups. Table 1 summarizes this evolution by contrasting the traditional rationale supporting each foundational assumption with the contemporary evidence that has led to its re-evaluation. Together, these findings illustrate how paracetamol has become a paradigmatic example of evidence reversal in medicine, whereby long-standing clinical beliefs have been reconsidered in light of more rigorous and comprehensive scientific data.

**Table 1:** The three foundational assumptions about paracetamol and their re-evaluation considering contemporary evidence

Assumption	Traditional basis	Contemporary findings
**Analgesic Efficacy**	Highly effective first line agent for acute/chronic pain [5]	*Limited or no benefit* Acute low back pain [8] Osteoarthritis [9] Chronic/Non-specific low back pain [10] *Established efficacy* Acute pain and fever [1]
**Safety Profile**	Lacks the gastrointestinal and platelet effects of NSAIDs and the dependence potential of opioids [5,20]	Leading cause of acute liver failure in overdose [3,13] Increased risk of cardiovascular, renal, and gastrointestinal events under chronic exposure [15,21]
**Clinical application**	First-line agent across age groups, in pregnancy and in multimorbidity [2,5]	Regulatory restrictions on dose and combination products [13] Debated neurodevelopmental concerns after prenatal exposure [22,23]

## Discovery and Early Pharmacological Development

**Fig. 1:**
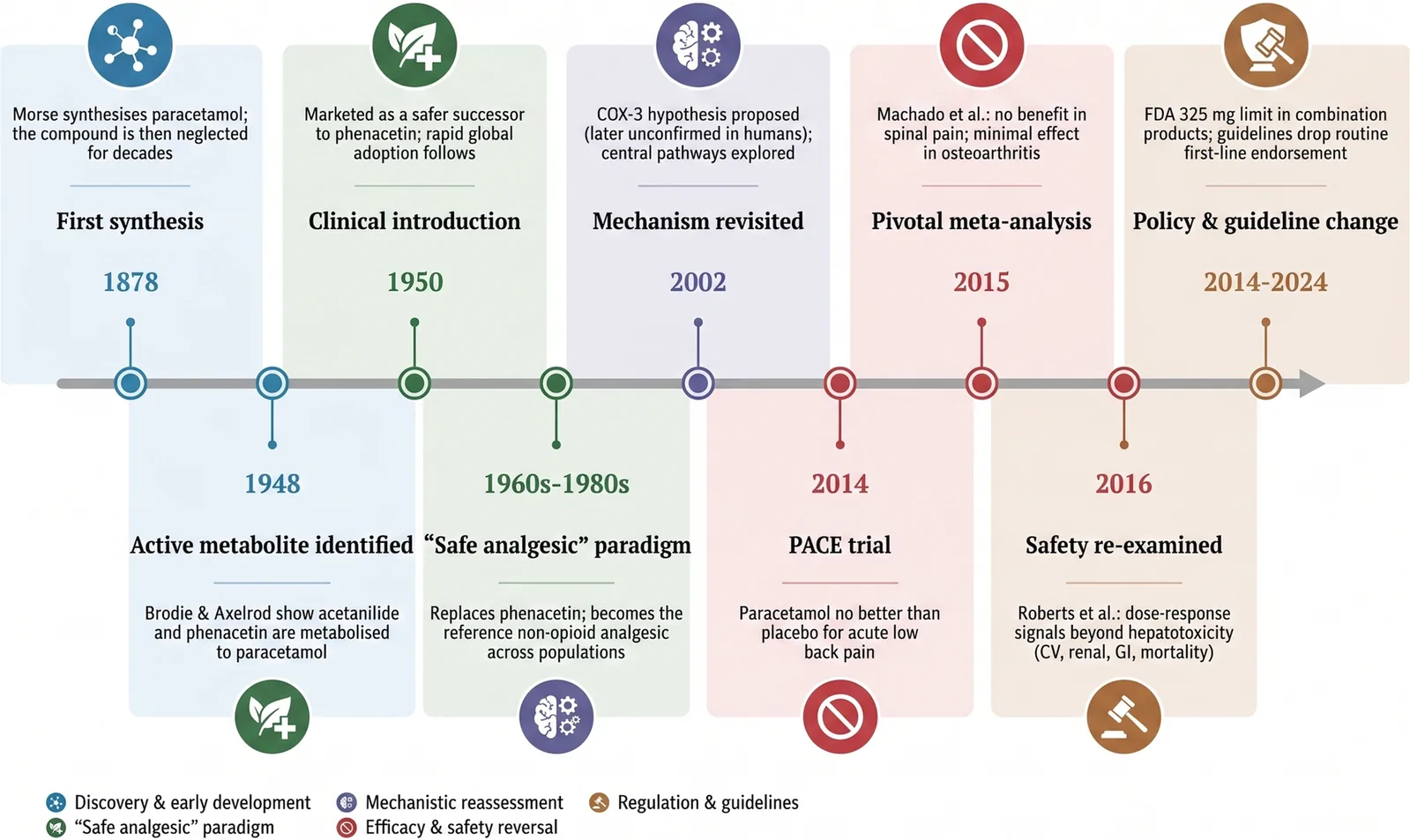
From discovery to evidence reversal: a timeline of the principal milestones in the history of paracetamol, from its synthesis to the randomized trials, safety analyses and regulatory actions of the past decade that prompted reassessment of its efficacy and safety-original figure [1, 5, 6, 8, 9, 11, 13, 15, 24–28]

The origins of paracetamol predate its clinical use by several decades and reflect the complex, often non-linear evolution of pharmacological knowledge (Fig. 1). The compound was first synthesized in 1878 by the American chemist Harmon Northrop Morse through the reduction of p-nitrophenol with tin in glacial acetic acid [24]. Despite its successful synthesis, paracetamol attracted little immediate scientific or therapeutic interest and remained largely overlooked during a period in which other aniline derivatives were rapidly gaining popularity as an antipyretic and analgesic agents [5].

At the end of the nineteenth century, acetanilide and subsequently phenacetin emerged as the dominant non-opioid analgesics. Early clinical investigations suggested that paracetamol possessed antipyretic properties but was associated with adverse hematological effects, a conclusion that contributed to the drug’s prolonged neglect and later proved erroneous [6]. Consequently, phenacetin became firmly established in clinical practice and remained widely used throughout the first half of the twentieth century.

The modern pharmacological history of paracetamol began only in the late 1940s, when Brodie et al. [25] demonstrated that both acetanilide and phenacetin were metabolized to p-hydroxyacetanilide, later identified as paracetamol, and that this metabolite accounted for much of their analgesic and antipyretic activity. These observations fundamentally altered prevailing pharmacological concepts and prompted renewed interest in paracetamol as a potentially safer therapeutic alternative. Subsequent clinical investigations confirmed its efficacy and relative tolerability, leading to its commercial introduction in the United States in 1950 and progressively throughout Europe during the following decade [6].

The emergence of paracetamol coincided with growing recognition of the nephrotoxicity and carcinogenic potential associated with chronic phenacetin exposure. As concerns regarding analgesic nephropathy intensified, paracetamol increasingly replaced its predecessors and gradually became regarded as the reference non-opioid analgesic [5]. This transition laid the foundation for the remarkable global expansion of paracetamol during the second half of the twentieth century and for its subsequent elevation to the status of one of the most widely used medications in modern medicine.

## Emergence as a Successor to Acetanilide and Phenacetin

The rise of paracetamol to clinical prominence was driven less by its initial therapeutic appeal than by the progressive recognition of the limitations of its predecessors. Growing evidence of hematological toxicity associated with acetanilide and of analgesic nephropathy, and urothelial malignancies linked to prolonged phenacetin exposure progressively undermined their clinical acceptance [5, 29].

As concerns regarding phenacetin toxicity intensified and regulatory restrictions expanded, paracetamol increasingly emerged as the preferred alternative. By the 1970s and 1980s, it had largely supplanted both acetanilide and phenacetin in clinical practice, establishing itself as the reference non-opioid analgesic in many countries [5, 6].

### Establishment of the “safe analgesic” Paradigm

The widespread acceptance of paracetamol during the second half of the twentieth century was founded not only on its perceived analgesic efficacy but also on its reputation as an exceptionally safe therapeutic option. As concerns regarding phenacetin-associated nephropathy and the gastrointestinal toxicity of NSAIDs became increasingly apparent, paracetamol was progressively positioned as the preferred first-line analgesic for a broad range of painful conditions [5]. Unlike NSAIDs, it was not associated with significant gastric ulceration or platelet inhibition at therapeutic doses, while lacking the dependence potential and other adverse effects traditionally associated with opioid analgesics [30]. Consequently, paracetamol came to be viewed as a uniquely versatile agent suitable for children, older adults, pregnant women, and patients with multiple comorbidities [7, 20, 31].

This favorable perception was further reinforced by decades of extensive clinical use, widespread over-the-counter availability, and inclusion in numerous national and international treatment guidelines. In many healthcare systems, paracetamol became the default analgesic against which alternative therapies were compared [32]. However, the emergence of this “safe analgesic” paradigm occurred largely before the contemporary era of large, randomized trials, pharmacovigilance databases, and rigorous benefit-risk assessment frameworks. As a result, the drug’s safety profile was often defined in relative rather than absolute terms, that is, as being safer than available alternatives rather than intrinsically devoid of risk [5].

Subsequent decades revealed a more nuanced reality. While paracetamol remains one of the safest analgesics when used appropriately, accumulating evidence regarding hepatotoxicity, overdose-related morbidity, and potential long-term adverse effects has challenged the notion of complete harmlessness [13, 15, 33]. The evolution of this perception represents one of the most instructive examples of how therapeutic reputation can become embedded in clinical practice and persist despite changing evidence.

### Evolution of Mechanistic Understanding

Few aspects of paracetamol pharmacology have generated as much uncertainty and scientific debate as its mechanism of action. Unlike NSAIDs, whose analgesic and anti-inflammatory effects are primarily mediated through peripheral cyclooxygenase (COX) inhibition, paracetamol exhibits only weak peripheral anti-inflammatory activity despite its antipyretic and supposedly well-established analgesic properties. For decades, this apparent paradox fueled the search for alternative mechanistic explanations and contributed to a succession of sometimes conflicting hypotheses [30].

**Fig. 2:**
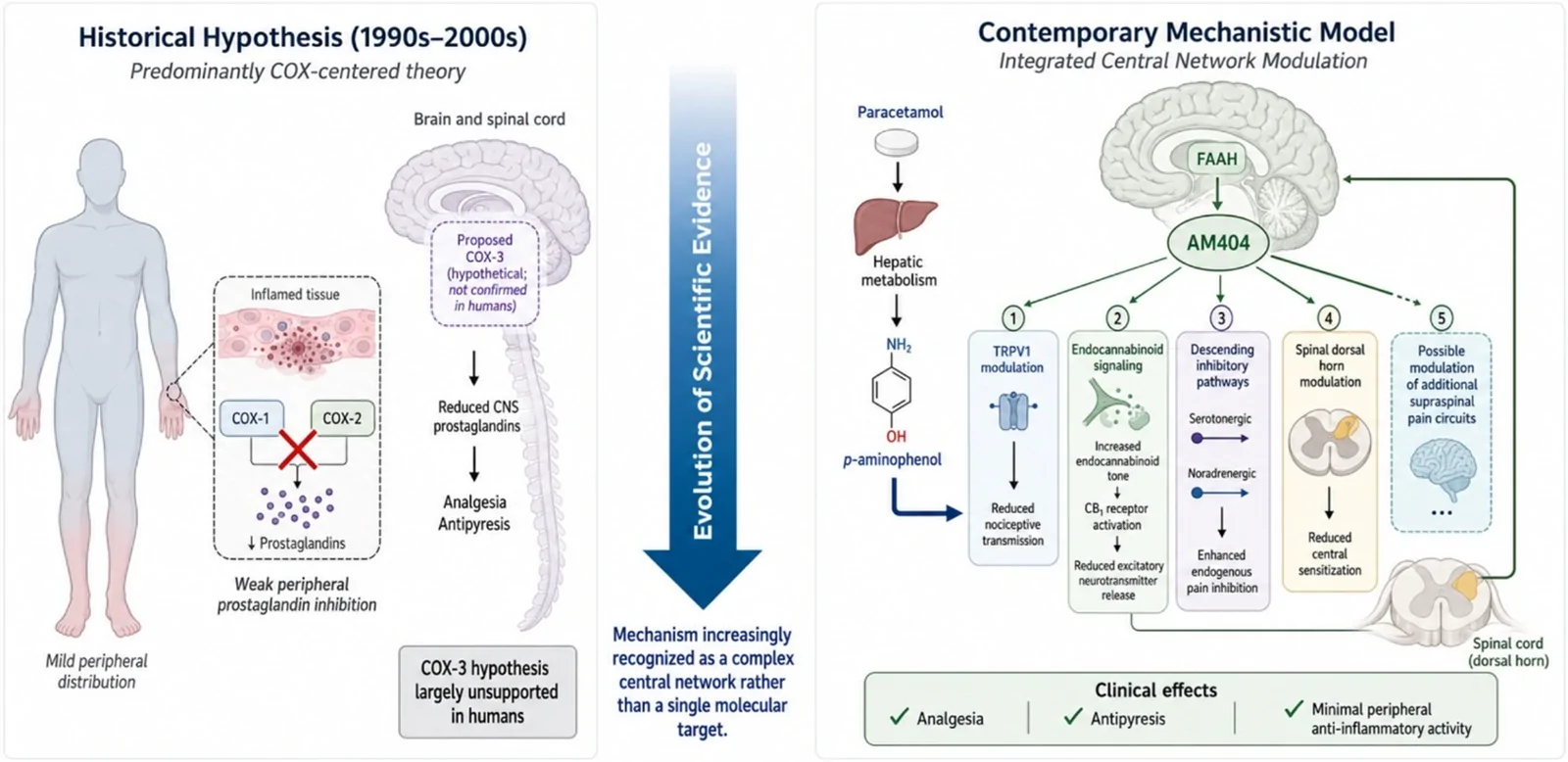
Evolution of the proposed mechanism of action of paracetamol. Early models attributed its analgesic and antipyretic effects to central inhibition of prostaglandin synthesis and a putative COX-3 isoform proposed in 2002, a single-target hypothesis subsequently unsupported in humans [26]. Contemporary models describe a multimodal central network: after systemic/hepatic metabolism to p-aminophenol and central conjugation with arachidonic acid via FAAH (Fatty Acid Amide Hydrolase) to form AM404 (N-arachidonoylphenolamine), paracetamol modulates endocannabinoid signaling through CB₁ receptors, TRPV1 channels and descending serotonergic pathways to produce its analgesic and antipyretic effects [34, 36, 37]- -original figure

Early investigations, including work by Flower and Vane in the early 1970’s suggested that paracetamol exerted its effects through central inhibition of prostaglandin synthesis, leading to the proposal of a putative COX-3 isoform as a specific molecular target. Although subsequent studies largely failed to confirm a clinically relevant role for COX-3 in humans, the concept stimulated broader exploration of central nervous system (CNS) mechanisms underlying paracetamol-induced analgesia [26]. More recent evidence indicates that paracetamol acts through a complex network of central pathways rather than a single molecular target [34, 35]. Following hepatic metabolism, paracetamol is converted to p-aminophenol, which subsequently undergoes conjugation with arachidonic acid in the CNS via the fatty acid amide hydrolase (FAAH) to form N-arachidonoylphenolamine (AM404). This metabolite has been implicated in the modulation of endocannabinoid signaling, transient receptor potential vanilloid 1 (TRPV1) channel, and descending serotonergic pathways, all of which contribute to analgesic processing [36, 37], (Fig. 2). This predominantly central mechanism of action provides a coherent explanation for the long-standing clinical observation that paracetamol relieves pain and fever while exerting little of the peripheral anti-inflammatory activity typical of NSAIDs. It also cautions against expecting NSAID-like efficacy in inflammatory musculoskeletal diseases.

Contemporary models therefore describe paracetamol as a centrally acting analgesic with multimodal mechanisms involving prostaglandin modulation, serotonergic neurotransmission, endocannabinoid signaling, and neurochemical pathways associated with pain perception. Although important questions remain unresolved, current understanding has moved far beyond the simplistic view of paracetamol as merely a weak cyclooxygenase inhibitor. The evolution of these mechanistic concepts illustrates how advances in molecular pharmacology can fundamentally reshape the interpretation of a drug that has been in clinical use for more than half a century [37, 38].

### Reassessment of Efficacy in Chronic Pain Conditions

Perhaps no aspect of its therapeutic profile has undergone a more profound reassessment than its role in chronic pain management. For several decades, paracetamol was widely recommended as a first-line treatment for a broad spectrum of chronic musculoskeletal disorders, largely owing to its favorable safety profile, low cost, and widespread availability. Its inclusion in numerous national and international guidelines was often based on long-standing clinical experience and extrapolation from acute pain models rather than on robust evidence derived from adequately powered randomized controlled trials [5].

This paradigm began to shift during the last decade as increasingly rigorous clinical investigations challenged assumptions regarding the magnitude and clinical relevance of paracetamol’s analgesic effects. A pivotal turning point was the PACE trial, a large randomized controlled study demonstrating that paracetamol was no more effective than placebo in reducing pain intensity, disability, recovery time, sleep quality, or quality of life in patients with acute low back pain [8]. Shortly thereafter, a comprehensive meta-analysis by Machado et al. [9] confirmed that paracetamol provided no meaningful benefit for spinal pain and only minimal, clinically insignificant improvements in pain and function among patients with osteoarthritis. Quantitatively, the pooled effect on low back pain was indistinguishable from placebo (weighted mean difference of approximately − 0.5 on a 0-100 pain scale), while the osteoarthritis benefit, although statistically significant, fell well below the threshold generally regarded as clinically important. These findings questioned the scientific basis of recommendations that had remained largely unchallenged for decades.

Subsequent evidence syntheses reinforced this reassessment. Contemporary reviews have consistently concluded that paracetamol offers limited or negligible efficacy in chronic low back pain and provides, at best, modest short-term symptomatic relief in selected patients with osteoarthritis [10, 39]. Therefore, several guideline committees revisited their recommendations, moving away from routine endorsement of paracetamol as a universal first-line analgesic and emphasizing a more individualized approach to pain management [39]. Importantly, these findings did not establish that paracetamol is ineffective in all chronic pain states; rather, they highlighted a discrepancy between historical therapeutic beliefs and the standards of evidence expected in contemporary medicine. The reassessment of paracetamol in chronic pain therefore represents a paradigmatic example of evidence reversal, whereby widely accepted clinical practices were reconsidered following the emergence of higher-quality evidence capable of challenging long-standing assumptions [40].

### Emergence of Safety Concerns Beyond Hepatotoxicity

Although hepatotoxicity remains the most recognized adverse effect of paracetamol, the last two decades have witnessed growing attention toward a broader spectrum of potential safety concerns associated with chronic or high-dose exposure. As the drug became increasingly used for long-term management of musculoskeletal pain, observational studies and pharmaco-epidemiological analyses began to challenge the long-standing perception of paracetamol as an essentially risk-free analgesic [41]. Accumulating evidence suggested possible associations between prolonged use and increased risks of cardiovascular events, hypertension, gastrointestinal complications, renal impairment, and overall mortality, especially at the upper range of recommended therapeutic doses [15, 42].

A landmark systematic review by Roberts et al. [15] demonstrated consistent dose-response relationships across several adverse outcomes, raising important questions regarding the assumption that paracetamol-related toxicity is confined to overdose or liver injury. Notably, several of these associations were graded with dose, with the highest risk estimates in patients exposed to the upper end of the therapeutic range, although the observational design precludes firm causal inference. Subsequent reviews reinforced concerns regarding chronic renal dysfunction, cardiovascular risk, and adverse outcomes associated with sustained exposure, while emphasizing the limitations inherent to observational data and the possibility of residual confounding [21, 42]. More recently, attention has expanded toward special populations, particularly pregnant women, following reports suggesting potential associations between prenatal paracetamol exposure and neurodevelopmental outcomes in offspring [16]. Although causality remains unproven and highly debated, these findings contributed to a more cautious interpretation of long-term safety.

Collectively, these developments have not overturned the favorable benefit-risk profile of paracetamol in appropriate clinical settings. Rather, they have transformed its safety narrative from one of presumed harmlessness to one requiring the same critical pharmacovigilance applied to other used analgesics [43]. The evolution of these concerns represents a further example of how therapeutic assumptions may be progressively redefined as more sophisticated epidemiological and clinical evidence becomes available.

### Regulatory Responses and Policy Changes

The evolving understanding of paracetamol efficacy and safety has been accompanied by a series of regulatory interventions aimed at reducing preventable harm while preserving access to an extremely widely used drug. Early regulatory attention focused primarily on overdose-related hepatotoxicity, which had emerged as a leading cause of acute liver failure in several countries [44, 45]. In response, multiple agencies introduced measures to improve risk communication, limit excessive dosing, and reduce unintentional overdose. Among the most influential actions was the decision of the United States (U.S) Food and Drug Administration (FDA) to restrict the amount of paracetamol contained in prescription combination products to 325 mg per dosage unit and to strengthen warnings regarding severe liver injury [13]. Similar initiatives were adopted in other jurisdictions, including limitations on pack sizes and enhanced labeling requirements intended to reduce overdose-related morbidity and mortality [46].

More recently, regulatory attention has expanded beyond hepatotoxicity to encompass broader questions regarding appropriate clinical use. The publication of large, randomized trials and meta-analyses demonstrating limited efficacy in low back pain and modest benefits in osteoarthritis contributed to revisions of several clinical practice guidelines. These bodies progressively shifted away from their long-standing historical recommendations, such as the initial endorsement of paracetamol as a standard first-line pharmacotherapy by the National Institute for Health and Care Excellence (NICE) in 2009 [47] and as a preferred oral default by the American College of Rheumatology (ACR) in 2012 [48]. This standard was explicitly reversed when the updated ACR/Arthritis Foundation guidelines downgraded paracetamol to a conditional recommendation in 2019 [28], reflecting a broader clinical transition toward restricted or individualized positioning prompted by contemporary meta-analyses (9,11; Table 2). These developments illustrate a broader shift from a regulatory paradigm centered predominantly on safety toward one that increasingly considers the balance between efficacy, safety, and public-health value.

The history of paracetamol regulation therefore provides an instructive example of how evolving scientific evidence can influence both drug policy and clinical recommendations, albeit often with substantial delay relative to the accumulation of evidence [13, 49]. This administrative shift is particularly evident when contrasting modern restrictions against the historical regulatory baseline, where the U.S. Food and Drug Administration (FDA) in 2010 traditionally permitted widespread clinical reliance via an unrestricted over-the-counter monograph framework [27], and European consensus frameworks similarly recommended the drug as an open, foundational first-line choice for osteoarthritis in 2003 (50; Table 2). All these evolutions are summarized in Table 2.

**Table 2:** Evolution of major international guidelines and regulatory bodies

Organization	Condition	Former Recommendations	Current Standing	Rationale for Change
**NICE (UK)**	Low Back Pain	First-line Pharmacotherapy [47]	Not Recommended	Absence of benefit over placebo in high-quality RCTs [11]
**ACR (USA)**	Osteoarthritis	Preferred First-line [48]	Conditionally Recommended	Limited effect size; safety concerns over chronic NSAIDs-free default [28]
**EULAR (EU)**	Knee osteoarthritis	Recommended First-Line [50]	Restricted / Individualized	Re-evaluation of the benefit-risk ratio in long-term management [21,40]
**FDA (USA)**	General Analgesia	Unrestricted Unit Dosing [27]	Mandatory Dose Limits	Targeted reduction of unintentional Acute liver failure via combination products [13]

### Persistence of Clinical use Despite Evolving Evidence

One of the most intriguing aspects of the paracetamol story is not the emergence of evidence challenging its efficacy in several chronic pain conditions, but rather the limited impact that such evidence has had on everyday clinical practice. Despite landmark randomized trials and systematic reviews demonstrating little or no clinically meaningful benefit in conditions such as low back pain and only modest effects in osteoarthritis, paracetamol continues to occupy a privileged position within healthcare systems worldwide [4, 9, 10, 18, 51], often serving as the default first-line standard before consideration of more complex strategies [18, 31]. Some related factors may explain this discrepancy. Paracetamol is perceived as safer than both NSAIDs and opioids in populations at increased risk of gastrointestinal, cardiovascular, renal, or opioid-related adverse events [31]. In addition, its long-standing presence in clinical guidelines has reinforced prescribing habits, and clinicians frequently favor it, even when its expected benefit is modest, because it is familiar, inexpensive, widely accessible, and perceived as comparatively safe, particularly when alternatives carry greater risks [52].

## Discussion

The present investigation highlights that the history of paracetamol is best understood as an example of how therapeutic reputations can become established before being rigorously tested and then persist even after the supporting evidence has evolved. The central question raised by this history is therefore not whether assumptions are correct, but why they persist and resist revision.

However, these explanations sit on top of deeper behavioral and structural determinants of medical practice. Therapeutic inertia favors the continuation of established behaviors even after their rationale has changed [53]. Cognitive tendencies, including anchoring, status-quo preference, and an asymmetric weighting of affirmative rather than disproving information, may reinforce familiar clinical defaults [54].

Negative findings diffuse slowly, often over years rather than months [55], and discontinuing an established treatment is a distinct behavioral task that a negative trial alone may not accomplish [56]. In short, negative evidence may exert less influence on clinical behavior than the potential positive evidence that originally established practice, making paracetamol a paradigmatic example of the evidence-practice gap rather than an isolated case [18, 57].

Interpreting these findings requires resistance to two opposite oversimplifications. The reversal does not suggest that paracetamol is ineffective, nor that it is inherently unsafe. Rather, a single global reputation has been applied to a drug whose benefit–risk balance depends strongly on indication, dose, and duration. For short-term treatment of acute pain and fever robust evidence continues to support both its efficacy and comparative safety [1, 2, 20].

The clinical tension arises during routine, prolonged use for chronic musculoskeletal pain where even modest adverse risks carry disproportionate weight, which may tip the clinical balance toward net harm [15]. The safety narrative followed a parallel paradigm shift. Severe toxicity has long been recognized, but it was historically dismissed as a distinct complication of acute overdose [12, 13]. That traditional assumption was dismantled by the emergence of clear dose-response safety signals associating prolonged therapeutic dosing with a chronic systemic harm that is not confined to overdose scenarios [15]. In both domains, limited evidence and broad clinical familiarity were interpreted as stronger proof of benefit and safety than was actually available, effectively transforming a lack of negative into an unearned assumption of absolute safety.

Several lessons extend beyond paracetamol itself. Its trajectory illustrates the phenomenon of evidence reversal, where established practices are challenged or revised as higher-quality data emerges. This is a process that does not imply that earlier decisions were irrational, but that reflects the evolving nature of scientific knowledge and the progressive refinement of methodological standards [58]. From this, three practical lessons follow. First, interventions effectively grandfathered into practice before the modern evidentiary era warrant scheduled re-evaluation, in the same way that newer therapies are now expected to demonstrate benefit; long-standing use should confer no permanent exemption from scrutiny [58, 59]. Second, claims of safety should always be expressed together with their comparator and their indication, because a relative judgement generalized into an absolute one is precisely the move that distorted the paracetamol record [5]. Third, the generation of evidence and its implementation are separate problems; dislodging a low-value practice requires active de-implementation strategies, not merely a revised sentence in a guideline [2, 56, 60, 61].

Considerable uncertainty persists, and identifying its limits is an important part of the paracetamol story. The mechanism of action remains incompletely defined. The contemporary model invoking the metabolite AM404, endocannabinoid and serotonergic signaling, and modulation of TRPV1 channels is well supported but has not been resolved into a single, human-validated molecular target [34, 35, 37]. The evidence on long-term harm is largely observational and therefore vulnerable to confounding by indication and channeling bias, so that the reported dose-response associations remain suggestive rather than firmly causal in the absence of long-duration randomized data [15, 21]. The most contested frontier is prenatal exposure and neurodevelopment. Early epidemiological associations and a widely publicized consensus statement urging precaution [16, 62] were substantially attenuated toward the null when familial confounding was addressed through sibling-control analysis in a cohort of nearly 2.5 million children [22], and a 2025 umbrella review of systematic reviews concluded that existing evidence does not clearly establish a link with autism or attention-deficit/hyperactivity disorder, which attributes earlier signals largely to unmeasured and familial confounding [23]. Yet residual uncertainty remains as randomized trials in pregnancy are contested [63], and the competing risks of untreated maternal fever and pain must be weighed against any putative harm, a tension that recent public and regulatory controversy has amplified rather than resolved [64]. A further open question is whether subgroups defined by adequate dosing, treatment duration, or acute indications might yet show benefits that the chronic-pain trials were not designed to detect. There is ample evidence to date that paracetamol provides benefits for headaches and both acute and chronic pain states. Side effects can be life-threatening and include potential adverse effects even in pregnancy [65–70].

The most durable lesson of the paracetamol story is epistemic rather than pharmacological. Better evidence did not make the drug less used, only more accurately understood. The same scrutiny can be applied prospectively to other familiar interventions, consistent with a view of evidence-based medicine as a dynamic, self-correcting process of continuous re-evaluation, not as a fixed body of knowledge. Consequently, the assumption that a long-used, well-tolerated drug is effective and safe across all its uses is best treated as a hypothesis to be tested rather than a settled fact [58, 59].

### Limitations

As with all narrative reviews, source selection and interpretation inevitably involve a degree of author judgment. Although systematic search methods were employed and SANRA principles were followed throughout the review process, the synthesis was designed to provide historical and conceptual insight rather than quantitative estimates of treatment effect. Consequently, the conclusions should be interpreted as an evidence-informed historical analysis rather than as a formal systematic review or meta-analysis.

A second set of limitations concerns the evidence being interpreted rather than the method of interpretation. Much of the data on long-term safety derives from observational studies that are susceptible to confounding by indication, channeling bias, and residual confounding, so that associations cannot be equated with causation. The efficacy trials differ in dose, duration, comparator, and population and predominantly address chronic musculoskeletal conditions, and therefore may not capture benefit in other settings or in adequately dosed subgroups. Finally, the rapidly evolving and contested literature on pregnancy and neurodevelopment means that the present balance of evidence should be expected to shift as further data accumulate.

## Conclusion

The story of paracetamol extends beyond pharmacology. It provides a case study in the sociology of medical knowledge and the evolution of evidence-based medicine. It demonstrates how clinical assumptions become institutionalized, how scientific paradigms can persist despite contradictory findings, and how healthcare systems struggle to adapt when long-held beliefs are challenged. Ultimately, the history of paracetamol serves as a reminder that no therapeutic intervention should be considered immune to reassessment. In medicine, certainty is often temporary, and even the most familiar drugs may reveal unexpected complexities when subjected to rigorous scientific scrutiny.

## Key References

- Williams CM, Maher CG, Latimer J, McLachlan AJ, Hancock MJ, Day RO, et al. Efficacy of paracetamol for acute low-back pain: a double-blind, randomised controlled trial. Lancet. 2014;384(9954):1586-96.
  - outstanding double blind randomised controlled trial on paracetamol for low-back pain.

- Roberts E, Delgado Nunes V, Buckner S, Latchem S, Constanti M, Miller P, et al. Paracetamol: not as safe as we thought? A systematic literature review of observational studies. Ann Rheum Dis. 2016;75[3]:552-9.
  - Excellent systematic review focused on safety of paracetamol.

- Orandi BJ, McLeod, MC, MacLennan PA, Lee WM, Fontana, RJ, Karvellas, CJ, et al. Association of FDA Mandate Limiting Acetaminophen (Paracetamol) in Prescription Combination Opioid Products and Subsequent Hospitalizations and Acute Liver Failure. JAMA. 2023;329[9]:735 − 44.
  - Important mandate from the FDA on acetaminophen with opioid product combination.

## Supplementary Information

Below is the link to the electronic supplementary material.

Supplementary Material 1

Supplementary Material 2

Supplementary Material 3

Supplementary Material 4

Supplementary Material 5

Supplementary Material 6

Supplementary Material 7

Supplementary Material 8

## Data Availability

No datasets were generated or analysed during the current study.

## Abbreviations

Abbreviation: Full Term
ACR: American College of Rheumatology
ADHD: Attention-Deficit/Hyperactivity Disorder
AM404: N-Arachidonoylphenolamine
CB_1_: Cannabinoid Receptor Type 1
CNS: Central Nervous System
COX: Cyclooxygenase
EULAR: European Alliance of Associations for Rheumatology
FAAH: Fatty Acid Amide Hydrolase
FDA: Food and Drug Administration
NICE: National Institute for Health and Care Excellence
NSAIDS: Nonsteroidal Anti-Inflammatory Drugs
RCTs: Randomized Controlled Trials
SANRA: Scale for the Assessment of Narrative Review Articles
TRPV1: Transient Receptor Potential Vanilloid 1
